# Impact of COVID-19 on the epidemiological features of mycoplasma pneumoniae infection in children with community-acquired pneumonia in Ganzhou, China

**DOI:** 10.3389/fimmu.2026.1765265

**Published:** 2026-04-07

**Authors:** Guihua Lai, Zhiyong Lai, Jungao Huang, Fucheng Peng, Qiying Gu

**Affiliations:** 1 Department of Medical Genetics, Ganzhou Maternal and Child Health Hospital, Ganzhou, China; 2 Clinical Laboratory, Ganzhou Maternal and Child Health Hospital, Ganzhou, China; 3 Department of Ultrasound Medicine, Ganzhou Maternal and Child Health Hospital, Ganzhou, China

**Keywords:** children, community-acquired pneumonia, COVID-19, epidemiology, immunity debt, mycoplasma pneumoniae

## Abstract

**Background:**

The COVID-19 pandemic and non-pharmaceutical interventions (NPIs) have altered the global epidemiology of respiratory pathogens, including *Mycoplasma pneumoniae* (MP). However, the impact of MP infection on children with community-acquired pneumonia (CAP) in Ganzhou, China, remains unclear. This study aims to investigate the influence of the COVID-19 pandemic on the epidemiology and clinical outcomes of MP infection among hospitalized pediatric CAP patients in Ganzhou, China.

**Methods:**

We retrospectively analyzed 27,369 hospitalized pediatric CAP patients (2017–2024), comparing MP positivity, seasonality, age distribution, and severity across pre-pandemic, NPIs, and post-NPIs phases.

**Results:**

Among 27,369 CAP patients, 3,334 were MP-positive (12.18%). The positivity rate dropped during Phase II (3.78%, 158/4,183) then rebounded sharply in Phase III to 21.17% (2,385/11,268), exceeding the pre-pandemic baseline (χ²=45.65, *p* < 0.001). The seasonal pattern exhibited changes, with the spring positivity rate increasing from 4.06% (146/3,594) in Phase I to 19.94% (577/2,893) in Phase III (χ²=505.33, *p* < 0.001). Regarding age distribution, the 7–10 years age group showed the highest positivity rate 30.76% (638/2,074; χ²=1,789.43, *p* < 0.001). However, in Phase III, the 4–6 years group exhibited a significant rise to 29.79% (883/2,964), compared with 13.00% (184/1,415) in Phase I and 7.87% (41/521) in Phase II (χ²=226.65, *p* < 0.001). In the 1–3 years group, the proportion of severe pneumonia reached its highest numerical proportion in Phase II (14.55%, 8/55), which was significantly greater than the proportions in Phase I (2.56%, 8/313) and Phase III (10.21%, 82/803; χ²=20.08, *p* < 0.001). Notably, both the median hospitalization duration and the rate of mechanical ventilation for severe pneumonia were significantly higher in Phase I than in Phases II and III.

**Conclusion:**

After the relaxation of NPIs, pediatric MP infections among hospitalized pediatric CAP patients in Ganzhou not only rebounded strongly but also exhibited a shift in epidemic peaks toward spring, alongside a notable change in susceptibility among younger children.

## 1 Background

Community-acquired pneumonia (CAP) is one of the leading causes of morbidity and mortality worldwide, particularly among children (1). Its epidemiological characteristics are complex and dynamically influenced by multiple factors including pathogen prevalence spectrum, seasonal variations, and geographical differences (2). Among these, *Mycoplasma pneumoniae* (MP) is a significant atypical pathogen of CAP, known for its regional cyclical epidemics occurring every 3–7 years in the pre-pandemic era (3). Non-pharmaceutical interventions (NPIs) like mask-wearing and social distancing, employed globally to combat COVID-19, dramatically altered the transmission patterns of respiratory diseases worldwide (4). These actions not only helped slow COVID-19 but also drove many common respiratory pathogens to historically low levels (5). This period of reduced pathogen circulation may have led to an accumulation of susceptible individuals, creating a phenomenon often referred to as ‘immunity debt’ (6). Following the gradual lifting of NPIs in China by late 2022, multiple regions reported a significant resurgence of respiratory infections. Notably, the timing, epidemic intensity, and clinical severity of MP infections exhibited unusual variations (7, 8). Currently, There was a lack of systematic research elucidating the epidemiological trajectory of MP infection in CAP during the COVID-19 pandemic in Ganzhou. Clarifying this change holds significant implications for clinical diagnosis, treatment, and the allocation of public health resources for pediatric MP infections in this region. This study aims to quantify the NPI-driven seasonal variations of MP and shifts in age-specific disease burden through longitudinal surveillance from 2017–2024, with particular focus on its implications for the ‘immunity debt’ phenomenon.

## 2 Materials and methods

### 2.1 Study population and design

This study analyzed the epidemiological data of CAP at Ganzhou Maternal and Child Health Hospital from January 2017 to December 2024. The study subjects were patients initially diagnosed with CAP, which was diagnosed by pediatricians according to WHO guidelines (9). Severe pneumonia was defined according to Chinese pediatric CAP management guidelines (10), specifically as cases presenting with multilobar involvement or involvement of ≥2/3 of a single lung lobe on chest imaging. The disease burden of severe pneumonia was assessed by examining clinical outcomes, including hospitalization duration and the need for invasive mechanical ventilation. The study protocol was approved by the Institutional Ethics Committee of Ganzhou Maternal and Child Health Hospital. Inclusion criteria included: 1) hospitalized children under 18 years old diagnosed with CAP; 2) availability of MP real-time polymerase chain reaction test results; and 3) availability of complete electronic medical records containing key clinical data, including hospitalization duration, imaging results, need for invasive mechanical ventilation, age, and sex. Exclusion criteria included: 1) Children with severe congenital or acquired diseases, including but not limited to: known immunodeficiency disorders, significant congenital heart disease, and congenital airway abnormalities; 2) Those receiving immunosuppressive therapy or patients with active malignant tumors. Seasonal variation was analyzed using meteorological definitions: spring (March–May), summer (June–August), autumn (September–November), and winter (December–February). To assess pandemic impacts, the study period was divided into three phases based on China’s COVID-19 policy adjustments: pre-pandemic (Phase I: 2017–2019), NPIs implementation (Phase II: 2020–2022), and post-NPIs (Phase III: 2023–2024). The age stratification is as follows: 0–1, 1–3, 4–6, 7–10, and 11–18 years old.

### 2.2 Specimen collection and detection

Trained medical professionals collected nasopharyngeal swabs from enrolled children and immediately transported them to the central clinical molecular laboratory. The testing was performed using DaAn Gene Co., Ltd. of Sun Yat-sen University’s MP nucleic acid detection kit, employing TaqMan PCR technology with primers and probes targeting 16S rRNA for MP detection. MP PCR testing was routinely performed for all hospitalized patients with CAP throughout the study period (2017–2024), including during the COVID-19 pandemic. All procedures were strictly conducted by professionals in accordance with standard operating protocols (11).

### 2.3 Statistical analysis

Statistical analyses were performed using SPSS (version 26.0; IBM Corp.) and R software (version 4.3.3; R Foundation for Statistical Computing). The MP positivity rate, defined as the percentage of MP-positive cases among all tested patients with community-acquired pneumonia, was calculated for the overall cohort and across subgroups stratified by age, gender, season, and the three distinct phases of the COVID-19 pandemic. Continuous variables (e.g., age, length of hospital stay) are presented as median and interquartile range M (IQR), while categorical variables (e.g., gender, age group, season) are summarized as frequencies and percentages. Group comparisons were conducted using the Kruskal-Wallis test for continuous variables and the chi-square test for categorical variables. For *post-hoc* pairwise comparisons following a significant overall test result, p-values were adjusted using the Bonferroni method to control for multiple testing. A corrected *p*-value < 0.05 was considered statistically significant. To further examine the potential nonlinear relationship between age and MP positivity rate, a multivariable logistic regression model was fitted using restricted cubic splines (RCS). In this model, MP infection status served as the dependent variable, age (continuous) was included as the primary predictor, and adjustments were made for gender and season. Four knots were placed at the 5th, 35th, 65th, and 95th percentiles of the age distribution. The RCS analysis was implemented in R.

## 3 Result

### 3.1 Patient demographic characteristics and overall trend of MP infection

**Figure 1:**
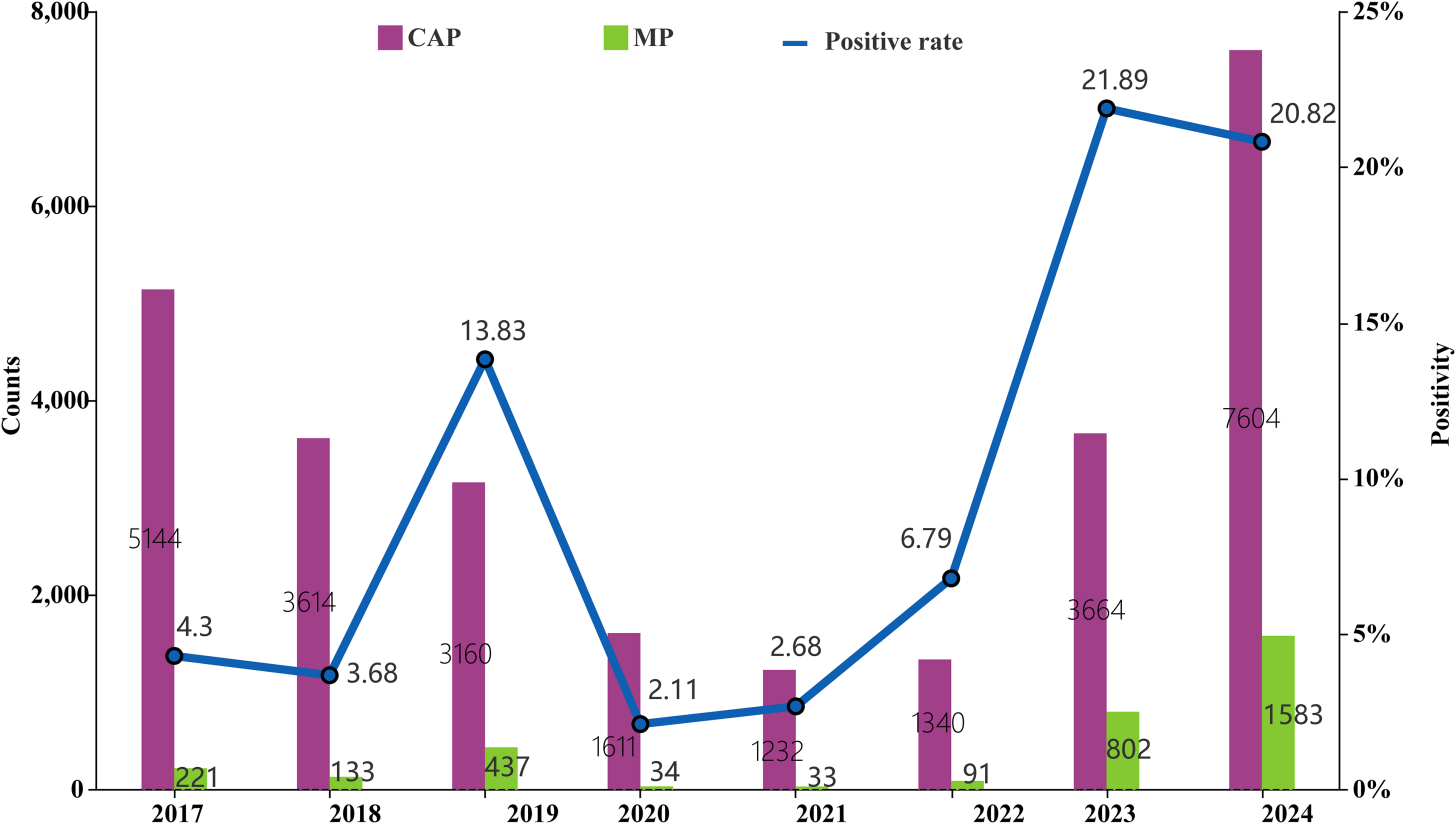
Trends in CAP hospitalizations and MP positivity rate among hospitalized CAP patients (2017–2024).

Among the 27,369 hospitalized children with CAP enrolled between January 2017 and December 2024, a total of 3,334 MP infections were detected, yielding an overall positivity rate of 12.18% (3,334/27,369). The median age of MP-positive children was 4 years (IQR: 2–6 years), with a slightly higher proportion of males (59.84%, 1,995/3,334) than females (40.16%, 1,339/3,334; **Table 1**). Following the COVID-19 outbreak, significant phase-dependent variations were observed in both CAP hospitalization numbers and MP positivity rates (**Figure 1**). The annual average number of CAP hospitalizations during Phase III was significantly higher than that in Phase I and Phase II. Moreover, there were significant differences in MP positivity rates across different phases (χ² = 45.65, *p* < 0.001). Additionally, the MP positivity rate also varied significantly across different age groups (χ² = 1,789.43, *p* < 0.001).

**Table 1:** Demographic characteristics of pediatric patients hospitalized with CAP.

Category	CAP n=27,369 (%)	MP n=3,334 (%)	Positive rate (%)	χ²	*p*
Male	16,950 (61.93)	1,995 (59.84)	11.77	6.96	<0.05
Female	10,419 (38.07)	1,339 (40.16)	12.85		
Phase I (SD)	3,973 (1039)	264 (156)	6.64	45.65	<0.001
Phase II (SD)	1,394 (195)	53 (33)	3.78		
Phase III (SD)	3,756 (2786)	818 (552)	21.17		
<1 year	8966 (32.76)	339 (10.17)	3.78	1,789.43	<0.001
1–3 years	10951 (40.01)	1171 (35.12)	10.69		
4–6 years	4900 (17.90)	1108 (33.23)	22.61		
7–10 years	2074 (7.58)	638 (19.14)	30.76		
11–18 years	478 (1.75)	78 (2.34)	16.32		

### 3.2 Evolution of seasonal epidemic patterns of MP infection

MP exhibited significant phase-dependent seasonal variations (**Supplementary Table 1**). During Phase I (2017–2019), a summer predominance was observed. Despite annual fluctuations, the MP positivity rate consistently remained high in summer, which was a distinctive feature of this phase, with the summer positivity rate in 2019 reaching 28.70% (283/986; χ²=282.81, *p* < 0.001). In 2020, the predominant period was Winter at 4.63% (15/324); in 2021, the main period shifted to Summer at 2.82% (8/284). Notably, a relative increase emerged in 2022, reaching 18.43% (61/331; χ²=79, *p* < 0.001). The third phase (2023–2024) is characterized by a strong rebound in epidemic intensity. The positivity rate surged sharply from 5.02% (12/239) in spring 2023 to 28.16% (548/1,946) in summer 2024. Meanwhile, the summer positivity rates remained high during 2023–2024(χ²=77.4, *p* < 0.001). Overall, from Phase I to Phase III, the MP positivity rate in summer increased from 15.50% (408/2,632) to 27.59% (689/2,497) (χ²=215.29, *p* < 0.001). More strikingly, the positivity rate in spring surged from 4.06% (146/3,594) in Phase I to 19.94% (577/2,893) in Phase III (χ²=505.33, *p* < 0.001) (**Supplementary Table 2**).

### 3.3 Changes in the age distribution of MP infection across different epidemic phases

**Figure 2:**
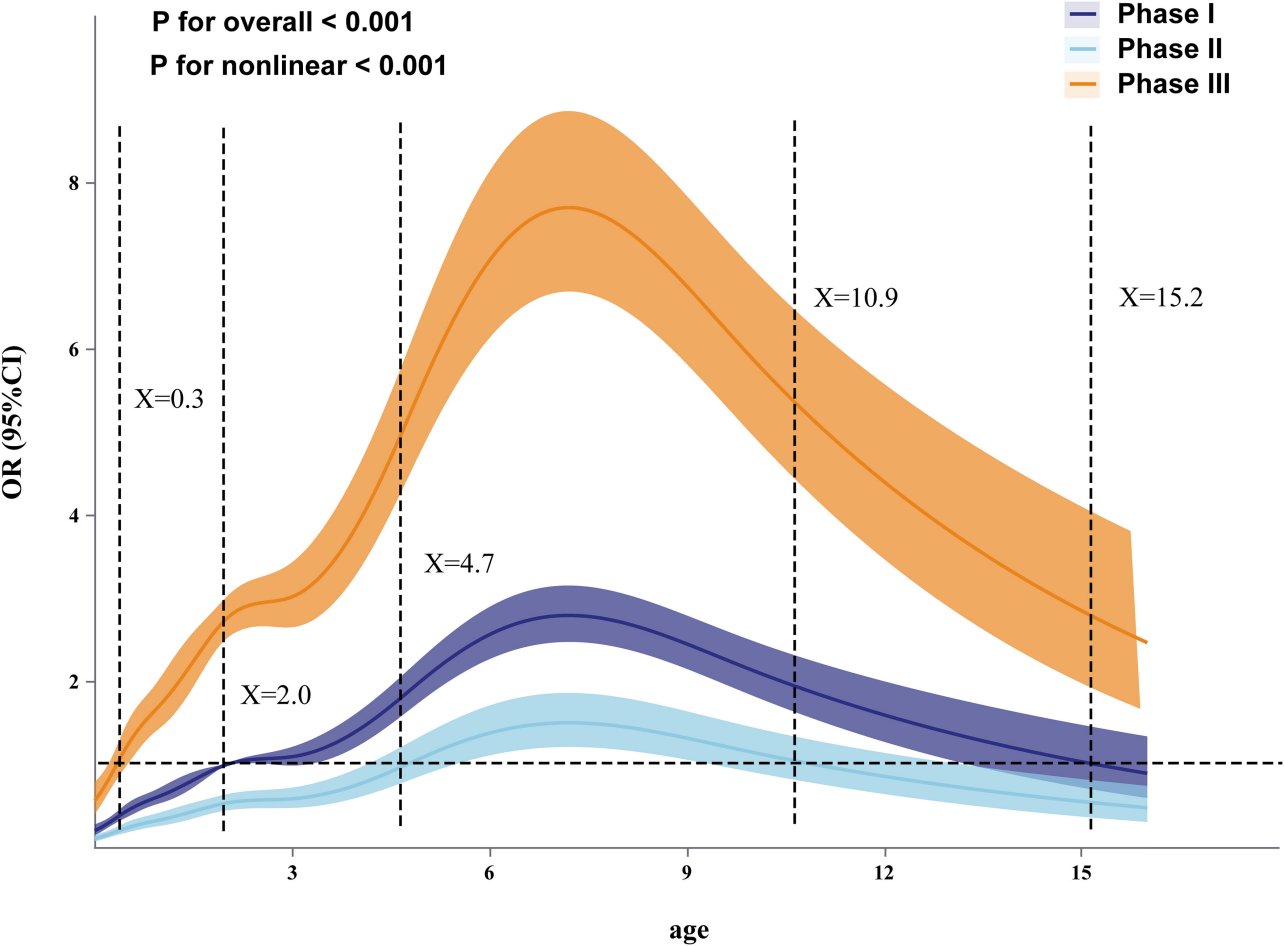
Nonlinear relationship between age and MP infection risk trends in three stages.

The MP positivity rate showed significant differences between age groups (**Table 2**). During Phase I, the 7–10 years group had the highest positivity rate (23.14%, 112/484; χ²=417.84, *p* < 0.001). In Phase III, although the positivity rate in this group further increased to 35.97% (500/1,390; χ²=60.53, *p* < 0.001), the 4–6 years group exhibited a more pronounced rise, reaching 29.79% (883/2,964; χ²=226.65, *p* < 0.001). The positivity rate among younger children rebounded during Phase III. Compared with Phase I, the MP positivity rate in children aged 1–3 years increased from 6.66% (313/4,702) to 17.15% (803/4,681; χ²=369.64, *p* < 0.001). Using RCS regression, we found a significant nonlinear association between age and MP infection risk across the three pandemic phases (P for nonlinearity < 0.001; **Figure 2**). In Phase I, the odds ratio (OR) was >1 for patients aged 2 to 15.2 years. In Phase II, the risk decreased significantly across all ages, with an OR >1 only in those aged 4.7–10.9 years. In Phase III, a widespread, sharp rebound occurred; the OR was >1 for patients aged 0.3–16 years, exceeding the Phase I baseline.

**Table 2:** Changes in MP positivity rates across age groups during different phases.

Age	Phase I	Phase II	Phase III	χ²	*p*
<1 year	3.15% (163/5,177)	1.95% (35/1,798)	7.08% (141/1,991)	81.95	<0.001
1–3 years	6.66% (313/4,702)	3.51% (55/1,568)	17.15% (803/4,681)	369.64	<0.001
4–6 years	13.00% (184/1,415)	7.87% (41/521)	29.79% (883/2,964)	226.65	<0.001
7–10 years	23.14% (112/484)	13.00% (26/200)	35.97% (500/1,390)	60.53	<0.001
11–18 years	13.57% (19/140)	1.04% (1/96)	23.97% (58/242)	27.55	<0.001
χ²**^†^**	417.84	89.68	597.7		
*p* **^†^**	<0.001	<0.001	<0.001		

**^†^** The χ² and p-value in the last two rows test for differences across age groups within each phase. The χ² and p-value in the rightmost two columns test for differences across phases within each age group.

### 3.4 Age-stratified analysis of MP infection severity

**Figure 3:**
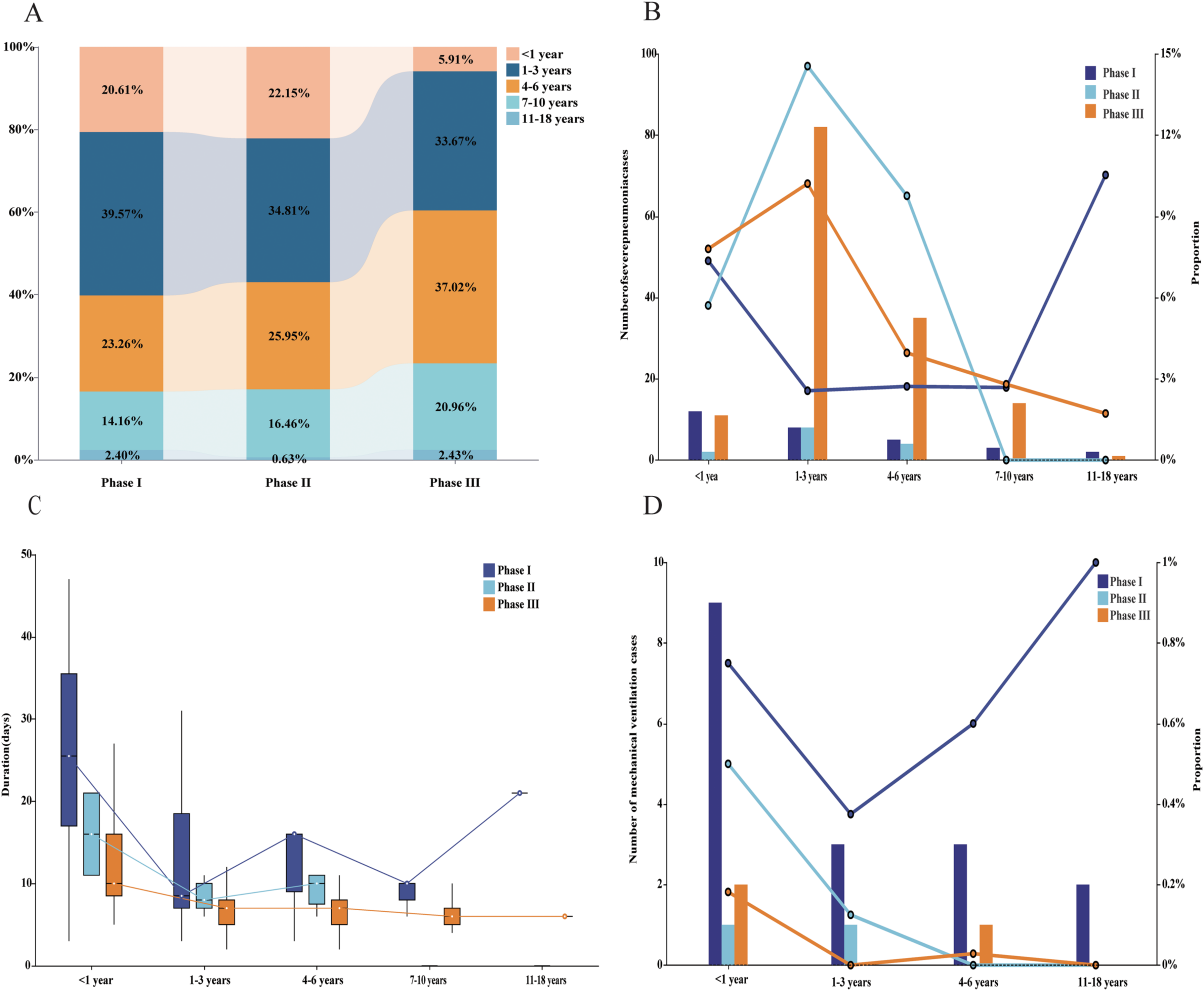
Age-Stratified Epidemiological Features of MP Infections Over 8 Years. **(A)** Age distribution of MP-infected children across pandemic phases. **(B)** Proportion and cases of severe pneumonia among MP-positive children by age group. **(C)** Hospitalization duration for severe MP-associated pneumonia by age group. **(D)** Proportion and cases requiring mechanical ventilation in severe pneumonia patients by age group.

Age-stratified analysis revealed significant differences in the age distribution of MP infection across all three phases (**Figure 3A**; **Supplementary Table 3**). Specifically, in Phase I, the 1–3 years age group accounted for the highest proportion (39.57%, 313/791; χ²=291.8, *p* < 0.001), while in Phase III, the dominant age group shifted to 4–6 years (37.02%, 883/2,385; χ²=1174.21, *p* < 0.001). Furthermore, the proportion of infants under 1 year old was 20.61% (163/791) in Phase I, which significantly decreased to 5.91% (141/2,385; χ²=149.58, *p* < 0.001) by Phase III. Regarding clinical severity, the proportion of MP-positive patients meeting the radiographic criteria for severe pneumonia varied by age and phase (**Figure 3B**). In the 1–3 years group, the proportion of severe pneumonia reached its highest numerical proportion in Phase II (14.55%, 8/55), which was significantly greater than the proportions in Phase I (2.56%, 8/313) and Phase III (10.21%, 82/803; χ²=20.08, *p* < 0.001). Similarly, in the 4–6 years age group, the proportion of severe pneumonia in Phase II was 9.76% (4/41), which appeared elevated compared to the 3.96% (35/883) in Phase III (χ²=1.98, *p*> 0.05). Among patients with severe MP pneumonia, the median hospitalization duration in Phase I was significantly longer than in Phase II and Phase III (**Figure 3C**). Further analysis of this severe pneumonia cohort revealed that the invasive mechanical ventilation rate in Phase I was also significantly higher than in the subsequent two phases (**Figure 3D**). Specifically, infants under 1 year old had the highest intubation rate in Phase I (75.00%, 9/12), which decreased to 18.18% by Phase III (2/11; χ²=7.43, *p* < 0.05). In the 1–3 years age group, the intubation rate decreased sharply from 37.50% (3/8) in Phase I and 12.50% (1/8) in Phase II to 0% (0/82; χ²=1.98, *p* < 0.001) in Phase III.

## 4 Discussion

This study systematically analyzed the dynamic impact of NPIs on the epidemiological characteristics of MP infection among hospitalized pediatric CAP patients, based on longitudinal surveillance data from 2017–2024. The results showed that during the NPIs implementation phase, the MP positivity rate significantly decreased. However, in the post-NPIs, the positivity rate exhibited a strong rebound, even surpassing the pre-pandemic baseline levels. This pattern of suppression followed by a vigorous rebound aligns with and provides support for the ‘immunity debt’ hypothesis (6). Prolonged NPIs effectively blocked the natural transmission of MP, resulting in a lack of immune exposure in the birth cohort during NPIs implementation, thereby accumulating a large susceptible population (12).

The implementation and lifting of NPIs also significantly altered the seasonal epidemic characteristics of MP infection. Pre-pandemic, MP infection in this region exhibited a typical summer peak. During NPIs implementation, this seasonal pattern was disrupted, manifesting as a low-level, non-seasonal sporadic mode, consistent with trends observed in the Netherlands, Germany, and other regions (13, 14). With the lifting of NPIs, MP infection not only re-emerged with a summer peak but also showed a significant increase in spring positivity rates, markedly expanding the epidemic time window. This change likely reflects the accumulation of a susceptible population and dynamic adjustments in pathogen-host interactions (15). Additionally, the age distribution and age-specific risk of MP infection have undergone significant shifts. Although school-aged children (7–10 years) remained the highest-risk group across all stages, the infection risk among preschool children (4–6 years) and adolescents (11–18 years) surged dramatically in the post-NPIs, becoming the predominant demographic groups in post-NPIs case composition. This shift in demographic structure further corroborates the ‘immunity debt’ effect (16). The concentrated infection of previously unexposed children has altered the traditional age distribution pattern dominated by school-aged children in the pre-pandemic era. Consistent with previous findings, the risk of MP infection among adolescents following the relaxation of NPIs was significantly elevated compared to other respiratory pathogens (17). In our cohort, MP infections accounted for 23.97% (58/242) of cases in this age group during the post-NPIs, further supporting these observations.

In terms of clinical severity, children aged 1–6 years showed an abnormal increase in severe cases during NPIs implementation. It should be noted that our study excluded pediatric cohorts with high-risk underlying conditions. We speculate that these children experienced delayed first exposure due to NPIs, becoming ‘immunologically naive’ individuals whose initial infection might trigger more severe lower respiratory tract clinical manifestations (18). Despite the surge in the number of infections during the post-NPIs, the average hospital stay and mechanical ventilation rate for severe MP pneumonia were lower in Phases II and III compared to Phase I. This may be attributed to the ongoing optimization of clinical management and population-level prevention strategies (19–21). Nevertheless, these gains do not fully offset the overall disease burden resulting from the expanded infected population and the relatively increased risk of severe disease among young children.

This study has several limitations. First, the observed trends in MP positivity rates may have been confounded by factors unrelated to pathogen circulation, such as changes in healthcare-seeking behavior or hospital admission policies during the NPIs period. Second, the sample size in Phase II was limited, which reduces the statistical power and precision for age-stratified comparisons, particularly in severity analyses.

Third, the absence of systematic data on co-infections with other respiratory pathogens limits our ability to assess their impact on MP incidence and disease severity. The co-circulation of viruses such as respiratory syncytial virus and influenza during the post-NPIs may have contributed to increased hospitalizations and shifts in the proportion of cases classified as severe (22, 23). Fourth, the lack of MP genotyping and macrolide-resistance testing precludes analysis of how pathogen genetics and antimicrobial resistance influence clinical outcomes (24, 25).Given the increasing prevalence of macrolide-resistant MP in China, such resistance could influence disease course and clinical management. Although not directly evaluated, improved clinical awareness and therapeutic strategies regarding macrolide-resistant MP over time may have contributed to the observed reductions in hospitalization duration and mechanical ventilation rates in severe cases (26). Finally, as a single-center study conducted in Ganzhou, our findings may not be generalizable to broader community-level MP transmission dynamics and thus require validation through multi-center or population-based studies.

## 5 Conclusion

Following the lifting of NPIs, MP infections among hospitalized pediatric CAP patients in Ganzhou rebounded vigorously, exhibiting an expanded spring-summer epidemic peak and a shift in susceptibility towards a bimodal pattern involving both preschool and school-aged children. This indicates that the ‘immunity debt’ induced by NPIs requires a period of immune landscape readjustment, and may chronically alter the epidemic intensity and temporal characteristics of MP.

## Data Availability

The datasets analyzed for this study are available at the request due to institutional policy on data curation. Requests to access these datasets should be directed to GQ, 1532992984@qq.com.
