# Supplementary material for: Impact of COVID-19 on the epidemiological features of mycoplasma pneumoniae infection in children with community-acquired pneumonia in Ganzhou, China
Source: Front Immunol. 2026 Apr 7;17:1765265. doi: 10.3389/fimmu.2026.1765265 (PMC13095516; doi:10.3389/fimmu.2026.1765265)
Supplement: Supplementary file 1 [file Table1.doc]

**Supplementary Table S1** Seasonal trends in MP positivity rate, 2017-2024.

| Seasons | 2017 | 2018 | 2019 | 2020 | 2021 | 2022 | 2023 | 2024 | χ² | *p* |
| --- | --- | --- | --- | --- | --- | --- | --- | --- | --- | --- |
| Spring | 2.40% (39/1,622) | 2.95% (36/1,221) | 9.45% (71/751) | 2.44% (4/164) | 1.36% (4/295) | 3.48% (14/402) | 5.02% (12/239) | 21.29% (565/2,654) | 600.38 | <0.001 |
| Summer | 6.81% (74/1,087) | 9.12% (51/559) | 28.70% (283/986) | 1.76% (6/340) | 2.82% (8/284) | 18.43% (61/331) | 25.59% (141/551) | 28.16% (548/1,946) | 431.56 | <0.001 |
| Autumn | 7.27% (72/991) | 3.91% (20/512) | 8.69% (63/725) | 2.49% (15/602) | 1.86% (6/323) | 4.94% (12/243) | 23.36% (459/1,965) | 9.93% (106/1,068) | 407.19 | <0.001 |
| Winter | 2.28% (56/2,460) | 2.98% (25/838) | 0.92% (5/541) | 4.63% (15/324) | 2.00% (8/400) | 1.04% (2/192) | 21.61% (549/2,540) | 1.88% (4/213) | 780.92 | <0.001 |
| χ²**^†^** | 81.2 | 41.46 | 282.81 | 5.62 | 1.63 | 79 | 46.72 | 187.37 |  |  |
| *P* **^†^** | <0.001 | <0.001 | <0.001 | >0.05 | >0.05 | <0.001 | <0.001 | <0.001 |  |  |

Footnote: For seasonal analysis, periods are defined as: Spring (Mar.–May.), Summer (Jun.–Aug.), Autumn (Sep.–Nov.), Winter (Dec.–Feb.).
Note: **^†^** The χ² and p-value in the last two rows of the table test for differences across seasons within each calendar year. The χ² and p-value in the rightmost two columns test for differences across years within each season.
