# Supplementary material for: Impact of COVID-19 on the epidemiological features of mycoplasma pneumoniae infection in children with community-acquired pneumonia in Ganzhou, China
Source: Front Immunol. 2026 Apr 7;17:1765265. doi: 10.3389/fimmu.2026.1765265 (PMC13095516; doi:10.3389/fimmu.2026.1765265)
Supplement: Supplementary file 2 [file Table2.doc]

**Supplementary Table S2 Seasonal trends in MP positivity rate, 2017-2024.**

| Seasons | Spring | Winter | Autumn | Summer | χ² | *p* |
| --- | --- | --- | --- | --- | --- | --- |
| Phase I | 4.06% (146/3,594) | 2.24% (86/3,839) | 6.96% (155/2,228) | 15.50% (408/2,632) | 503.79 | <0.001 |
| Phase II | 2.56% (22/861) | 2.73% (25/916) | 2.83% (33/1,168) | 7.85% (75/955) | 49.96 | <0.001 |
| Phase III | 19.94% (577/2,893) | 20.09% (553/2,753) | 18.63% (565/3,033) | 27.59% (689/2,497) | 77.4 | <0.001 |
| χ²**^†^** | 505.33 | 681.83 | 278.23 | 215.29 |  |  |
| *P* **^†^** | <0.001 | <0.001 | <0.001 | <0.001 |  |  |

Footnote: For seasonal analysis, periods are defined as: Spring (Mar.–May.), Summer (Jun.–Aug.), Autumn (Sep.–Nov.), Winter (Dec.–Feb.).
Note: **^†^**The χ² and p-values in the bottom two rows assess differences among pandemic phases within each season. The χ² and p-values in the rightmost two columns evaluate differences among seasons within each pandemic phase.
