# Supplementary material for: Impact of COVID-19 on the epidemiological features of mycoplasma pneumoniae infection in children with community-acquired pneumonia in Ganzhou, China
Source: Front Immunol. 2026 Apr 7;17:1765265. doi: 10.3389/fimmu.2026.1765265 (PMC13095516; doi:10.3389/fimmu.2026.1765265)
Supplement: Supplementary file 3 [file Table3.doc]

**Supplementary Table S3 Age distribution of MP-infected children across pandemic phases．**

| MP infections | Phase I (n=791) | Phase II (n=158) | Phase III (n=2,385) | χ² | *p* |
| --- | --- | --- | --- | --- | --- |
| <1 year | 20.61% (163) | 22.15% (35) | 5.91% (141) | 149.58 | <0.001 |
| 1-3 years | 39.57% (313) | 34.81% (55) | 33.67% (803) | 5.89 | >0.05 |
| 4-6 years | 23.26% (184) | 25.95% (41) | 37.02% (883) | 36.5 | <0.001 |
| 7-10 years | 14.16% (112) | 16.46% (26) | 20.96% (500) | 15.00 | <0.001 |
| 11-18 years | 2.40% (19) | 0.63% (1) | 2.43% (58) | 2.07 | >0.05 |
| χ²**^†^** | 291.8 | 51.114 | 1174.21 |  |  |
| *P* **^†^** | <0.001 | <0.001 | <0.001 |  |  |

Note: **^†^**The χ² and p-value in the last two rows of the table test for differences in age distribution (proportion of cases in each age group) within each pandemic phase. The χ² and p-value in the rightmost two columns test for differences in the proportion of cases across pandemic phases within each specific age group.
